# A Preliminary Exploration of Factors Affecting a University Entrepreneurship Ecosystem

**DOI:** 10.3389/fpsyg.2021.732388

**Published:** 2021-09-30

**Authors:** Xuyan Wang, Xiaoyang Sun, Shuhua Liu, Chen Mu

**Affiliations:** ^1^Office of Development and Planning, Hangzhou Normal University, Hangzhou, China; ^2^Ningbo University of Finance and Economics, College of Humanities, Ningbo, China; ^3^Zhejiang University, College of Education, Hangzhou, China; ^4^Zhejiang Sci-Tech University, School of Art and Design, Hangzhou, China

**Keywords:** entrepreneurship, university entrepreneurship ecosystem, students, extracurricular activities, network, culture, leadership

## Abstract

This study examines the factors that affect the formation and operation of the university entrepreneurship ecosystem (UEE). Employing the case-study methodology, this research attempts to provide an evidence-based analysis of the existing theoretical framework of the UEE and verify the role of elements in it through empirical experience as described in semi-structured interviews with 33 respondents on and off an American university. Findings reveal that extracurricular activities, networks, entrepreneurial culture, and leadership have an important impact on the formation and operation of the UEE. Specifically, compared with formal courses, as important carriers, extracurricular activities have a more positive impact on the entrepreneurship of students. Different levels of networks can promote the circulation and exchange of resources. Culture is an important factor in forming and promoting individual entrepreneurial behavior and their agglomeration in the UEE. Clear vision and long-term commitments to entrepreneurship, namely leadership, play a leading role in the formation and development of a UEE.

## Introduction

As a powerful force for addressing global challenges (Fetters et al., 2010) and contributing to the economic future (Daniel et al., 2018), entrepreneurship has slowly demonstrated its charm since the beginning of the twenty-first century. There is an emerging trend that the entrepreneurship ecosystem (EE) has become a focus in the field of entrepreneurship research (Zacharakis et al., 2003; Isenberg, 2010; Malecki, 2011; Feldman and Zoller, 2012; Mason and Brown, 2014). The EE can be considered as an environment surrounding entrepreneurial activities. If the entrepreneurial environment around entrepreneurs is healthy, the output of start-ups can improve the economic and non-economic performance of the local society (Art, 2018). From the perspective of social and economic development, the research on the EE is of great significance. EE can serve not only as a catalyst for accelerating the economic progress of a stable economy but also as a driving force to rescue an economy facing a sharp decline (Hechavarria and Ingram, 2014).

In the EE, the educational organization, especially the university, is often seen as an important aspect or element of the EE by many scholars. Universities play an important role in cultivating entrepreneurs (Campanella et al., 2013). Isenberg (2011) saw education institutions as one of the important contents of the EE. George and Carlos (2013) regarded the major universities as catalysts of the EE. In addition, organizations such as the Organization for Economic Cooperation and Development and the National Women's Business Council also regard universities as an important element of entrepreneurship ecology. Some scholars directly pointed out that the university itself has formed a unique EE, and research on the university entrepreneurship ecosystem (UEE) has been done subsequently (Fetters et al., 2010; Miller and Acs, 2017). A well-structured UEE can provide rich resources and institutional support for campus entrepreneurs. To explore the nature of the UEE, the following research questions frame the study as follows: (1) What are the key factors that influence the formation and development of a UEE? and (2) What role do these factors play in the UEE?

## Entrepreneurship Ecosystem

The term ecosystem was derived from the field of ecology. It was originally proposed by Roy Clapham in 1930 and adopted by Arthur Tansley in his famous article “The Use and Abuse of Vegetational Concepts and Terms” (Tansley, 1935; Brush, 2014). In 1993, for the first time, James F. Moore introduced the term “ecosystem” into the world of business and coined the concept of the business ecosystem (Moore, 1933; Mason and Brown, 2014; Neumeyer and Corbett, 2017; Daniel et al., 2018; Freitas and Kitson, 2018). He believed that the development of business depends on a range of stakeholders interacting with each other, such as suppliers, producers, sellers, market intermediaries, investors, governments, consumers, and so on (Moore, 1933).

Subsequently, the concept of the EE has received a lot of attention from academia which emphasizes the need for a more holistic and dynamic approach (Mason, 2019). The EE enables individuals, companies, and communities to effectively unite and create economic wealth and prosperity (Prahalad, 2005). The initial research mostly focused on exploring the concept of EEs. For example, Carvalho et al. (2010) proposed that the EE includes a set of tangible and intangible resources and actors, which are characterized by interdependent relationships that produce important synergies. Hechavarria and Ingram (2014) proposed that the EE is defined as a group of interrelated entrepreneurial actors, entrepreneurial organizations, institutions, and entrepreneurial processes that formally and informally combine to mediate and manage performance in the local entrepreneurial environment. From a larger perspective, Nicotra et al. (2018) proposed that the EE is a combination of social, political, economic, and cultural factors in a region, supporting the development and growth of innovative and entrepreneurial enterprises.

In addition to the definition of the EE, some scholars have analyzed the supporting elements or pillars of the EE. Cohen (2005) outlined seven factors of the EE, including informal network, formal network, university, government, professional and support services, capital services, and talent pools. Isenberg (2011) pointed out that the EE consists of six domains, namely, policy, finance, culture, market, human capital, and support system. George and Carlos (2013) put forward nine pillars of the EE, namely, market openness, human capital, funding and financing, mentor consultation support system, rules and regulations, facilities, education and training, university catalysis, and culture. Aldrich et al. (1989) concluded that the EE is characterized by family businesses and role models, a diversified economy, a strong business infrastructure, available investment capital, a supportive entrepreneurial culture, and public policies that encourage entrepreneurship.

The building strategies and evaluation methods of an EE are also scattered in the research of some scholars. Lawlor (2014) raised the strategies to build an EE, including attaching importance to the development of individual businesses or small- and medium-sized enterprises, regional entrepreneurial activities should break out of urban boundaries and form a network of multi-city and multi-regional linkages; strengthen the projects and financial support, strengthen the cooperation between academic institutions and business groups, and reduce financial obstacles in the development of enterprises. Mason and Brown (2014) analyzed the concept, characteristics, and model of the EE and discussed the principles, methods, and evaluation methods of policies supporting the development of the EE. Stangler and Jordan (2015) proposed four indicators to measure the activity of the entrepreneurial ecosystem, namely, density, liquidity, connectivity, and diversity.

## University Entrepreneurship Ecosystem

The place is part of the EE concept. The environment and the nature of the place can be considered as key factors affecting the arrangements of entities, availability of resources, and the nature of processes and interactions therein. The place can be as large as a region, a city, or as small as a company or university (Daniel et al., 2018). In this study, as a place, the university not only can be an important supporting element of the EE (Carvalho et al., 2010; Audretsch and Link, 2017) but can also become the EE itself. The EE developed with a university campus as a “place” is called university-based entrepreneurship ecosystem (UBEE) (Fetters et al., 2010; Ruth, 2014; Art, 2018) or UEE (Brush, 2014; Miller and Acs, 2017; Suryanto, 2019). These different terms all refer to ecosystems developed on the campus of universities, which are part of a larger ecosystem (Rice and Habbershon, 2006; Fetters et al., 2010), and different from regional ecosystems due to the knowledge attributes of universities. The creating of the UEE is currently a hot topic. With the development of entrepreneurship education in the United States, many universities have formed entrepreneurial centers dedicated to creating EEs (Torrance, 2013; Schmidt and Molkentin, 2015). Research on EE is of great significance to enhance the entrepreneurial willingness and entrepreneurship rate of students. The cooperation between universities and industries, as well as their knowledge generation and technological development, promote the derivation of high-tech enterprises and the emergence of start-ups (Audretsch and Link, 2017). Thus, the UEE can enhance the regional EE and form an entrepreneurship cycle, which can create more jobs and stimulate economic vitality.

Similar to the research on the EE, scholars pay more attention to the connotation and elements of the UEE. Fetters et al. (2010) initially framed the discussion of UBEE and identified seven factors that contribute to the evolution of UBEE, namely, senior leadership vision and engagement sponsorship, strong programmatic and faculty leadership, sustained commitment over a long period of time, the commitment of substantial financial resources, commitment to continuing innovation in curriculum and programs, appropriate organizational infrastructure, and commitment to building the extended enterprise and achieving critical mass. By identifying the most highly regarded entrepreneurial universities outside the world, Ruth (2014) summarized seven factors that underpin these UEE, namely, institutions, culture, university leadership, university research capabilities, regional or government support, effective institutional strategies, and strong student entrepreneurial demand. Different from the previous two scholars, he pointed out that entrepreneurship education or courses are considered an important element of the UEE. Suryanto (2019) believed that the components of the UEE should include higher education council policies, policies of university leaders in courses, the existence of business incubation centers, downstream research products, lecturers who change the thinking of students, and collaboration with government agencies and bank institutions. Based on the above components, he also proposed five strategies for creating the UEE, namely, good course design, improving the teaching methods of entrepreneurship courses, acceleration of research downstream products, collaboration with other institutions, and awarding best entrepreneurship. Similarly, Art (2018) identified the components of the UBEE, including curriculum, extra-curricular, traditional technology transfer office, bridging mechanisms, resources and community engagement, and informal engagement. Brush (2014) believed that the internal ecosystem of entrepreneurship education is the core of the UEE, and she divided the internal entrepreneurship education ecosystem into three major areas (i.e., start-up courses, extracurricular activities, and research) and four dimensions (i.e., stakeholders, resources, facilities, and culture).

The UEE can provide available assets, liberty, and diversity while creating opportunities and fostering entrepreneurship and innovation (Miller and Acs, 2017). However, most of the existing studies focus on the concept and elements of EEs, portraying a lack of sufficient empirical study (Stam, 2015; Audretsch et al., 2018; Shwetzer et al., 2019), let alone the empirical study of UEE. In addition, the role of the network (Ter and Boschma, 2011) and the interaction of individual elements in EEs have not been fully explored (Motoyama and Watkins, 2014).

## Research Design and Methods

This study adopted the qualitative research methodology as it could gain a complex, detailed understanding of the issue and identify variables that can be measured (Creswell, 1994, 2007). Specifically, this research combines empirical study with literature study, and a case study at an American university (AU) in Los Angeles was conducted.

### Site Selection and Access

The place is an important factor in choosing the case. We chose the case of AU, an AU with a strong entrepreneurial atmosphere on campus for the following reasons. First, AU is a top university located in the United States which has carried out entrepreneurship education on the campus for more than 60 years. The United States has formed a mature entrepreneurship education system and has a good entrepreneurial atmosphere (Fang and Liu, 2006). American universities are ideal locations for studying the UEE. Second, Los Angeles, an entrepreneurial city where the AU is located, has the largest angel investment group in the United States. In 2016, Los Angeles-based start-ups received $6 billion in financing which has increased by six times compared with 2012. In addition to San Francisco and New York, Los Angeles has become the third-largest entrepreneurial village in the United States. Its rapidly growing high-tech entrepreneurial cluster, namely, Silicon Beach, has become a strong competitor to Silicon Valley. In recent years, the number of companies in the Los Angeles area has increased rapidly, and a large number of incubators, accelerators, and technology communities have continuously emerged which undoubtedly provide AU students with rich resources and a good entrepreneurial environment (Wang and Ye, 2018). Third, since 2010, the leadership of AU has been committed to creating an EE on campus and it has made outstanding achievements in entrepreneurship. According to the statistics of the American Association of Technology Management, AU ranks among the top three universities in North America in terms of the number of new ventures started each year, with an average annual number of 15–25 new technology-based enterprises, and its entrepreneurship rate is higher than that of all other California system institutions. In 2014, AU ranked in the top five on the 2014 list of the most entrepreneurial universities of Forbes (Chen, 2017).

In terms of gaining access, the first author went to AU from 2016 to 2017 and conducted 1-year field research on the topic.

### Data Collection Strategies and Sampling

This study uses the following strategies to collect data: semi-structured interviews, participatory observation, and document analysis. The interactive nature of interviews allows the researcher to study complex subjects that could hardly be investigated in depth through questionnaires (Carvalho et al., 2010). There are two ways to get the interview samples. The first way was to search for an AU-related entrepreneurship event and contact the organizer or guest of the event directly for an interview. The second way was to obtain the information of entrepreneurial activities or events on the campus or Los Angeles area through Internet or App, mainly Eventbrite and Meetup. Then, the first author would participate in these activities according to the schedule and find the interviewees on the events. The first author totally participated in more than 40 in-campus and off-campus activities related to entrepreneurship, including business meetings, networking, training camps, seminars, entrepreneurial counseling, pitch competitions, and so on. Most of the semi-structured interviews were conducted at the networking session of each event. The interviews vary from 30 to 60 min.

To gain a deeper understanding of the UEE, the author conducted semi-structured interviews with 33 student entrepreneurs, mentors, event organizers, and entrepreneurs in Los Angeles. This research purposely designed three different interview outlines based on the different types of interviewees as student entrepreneurs, event organizers or mentors, and entrepreneurs in Los Angeles. For student entrepreneurs, the interview outline aims to understand the motivations of campus entrepreneurs, the support they receive, and the challenges they face. For event organizers or mentors, the interview outline aims to understand the motivations of participating in the UEE. For entrepreneurs in Los Angeles, the interview outline aims to understand the development of the entire entrepreneurial environment and entrepreneurial ecosystem in Los Angeles. Table 1 shows the 33 interviewees in the semi-structured interviews who are organized and coded by their status as student entrepreneurs, event organizers or mentors, and entrepreneurs in Los Angeles.

**Table 1:** Code list of interviewees.

**Type**	**Description of Interviewees**				
**Student entrepreneurs**	**Code**	**Gender**	**Identity**	**Discipline**	**Entrepreneurship projects**
	S1	Female	Undergraduate	Language and computer	Internet sales
	S2	Male	Undergraduate	Finance	Preparing for business(music)
	S3	Male	Undergraduate	Computer	Computer science (virtual reality)
	S4	Male	Postdoc	Material Engineering	New material (battery)
	S5	Female	Postdoc	Material Engineering	New material (battery)
	S6	Female	doctoral student	Economics	Movie and TV animation
	S7	Male	alumni	Mathematics	Real estate
	S8	Male	Undergraduate	Mathematics	Preparing for business
	S9	Male	Undergraduate	Computer	Computer science (virtual reality)
	S10	Male	alumni	Business	Catering Services
	S11	Male	Undergraduate	Business	Finance
**Event organizers or mentors**	**Code**	**Gender**	**Identity**		**Activity**
	P1	Male	AU alumni/Entrepreneur/Guest Lecture		Seminar
	P2	Male	AU student/organizer		LA Hacks
	P3	Male	Incubator CEO		Career Counseling
	P4	Male	AUalumni/Campus Project Coordinator / Entrepreneur Mentor / Entrepreneur		Consulting Activity
	P5	Female	AU Undergraduate/organizer		Club Event
	P6	Female	AU Undergraduate/organizer		Club Event
	P7	Female	organizer		Entrepreneurship Bootcamp
	P8	Female	Guest Lecture/Entrepreneur		Entrepreneurship Meeting on Campus
	P9	Female	Guest Lecture/Executive Producer		Entrepreneurship Meeting on Campus
	P10	Female	Guest Lecture/Counselor		Entrepreneurship Meeting on Campus
	P11	Male	Guest Lecture/Entrepreneur		Entrepreneurship Meeting on Campus
	P12	Female	Guest Lecture/Incubator CEO		Entrepreneurship Meeting on Campus
	P13	Female	Guest Lecture/Entrepreneur		Entrepreneurship Meeting on Campus
	P14	Male	Professor/organizer		Pitch Competition
**Entrepreneurs in Los Angeles area**	**Code**	**Gender**	**Identity**		**Entrepreneurial Area**
	E1	Male	Serial Entrepreneur		High Tech
	E2	Male	AU alumni/Engineer		High Tech
	E3	Male	Entrepreneur/Mentor		High Tech
	E4	Male	Entrepreneur		Artificial Intelligence
	E5	Male	Business Consultant		Business
	E6	Male	Engineer		Software
	E7	Female	Entrepreneur		Film and Television Industry
	E8	Male	Counselor		Legal Business

The observational method has been used in this research to gain an in-depth understanding of the entrepreneurial activities on campus. Observations occurred at a variety of entrepreneurship-related activities on- and off-campus, including AU career development conference, The Road to Series A organized by Tech Fire, L.A. Tech Happy Hour, Fireside Chats organized by Startup AU, First Fridays @ AU TDG, Networking and Demo Day at the Coding Boot Camp at AU Extension, Startup Game Changers Panel, AU Staff Assembly Learn-at-Lunch, Women Founders Network CEO Roundtables, Y combinator at AU, Startup Labs workshop, WSGR Entrepreneurs Boot Camp, The Lowell Milken Institute-Sandler Prize for New Entrepreneurs, Wilbur K. Woo Greater China Business Conference, etc. During the participation in these activities, important information displayed by the speakers or guests was recorded in detail through formalized field notes. Examples of participant observation used in this study include the following: observing students and mentors in entrepreneurial events interacting with each other, hanging out and interacting with students, and mentors and businessmen in entrepreneurial events and networking parties.

Literature has been explored based on the information collected, and in turn, the newly acquired literature supports this research. The documents collected can provide the research a more panoramic picture and gain a deeper understanding of the topic.

## Findings

### Extracurricular Activities as Important Carriers

Many students interviewed reported that entrepreneurship or commercial courses have not met their needs. S6, a doctoral student in economics from another university in Los Angeles who is attending the events of AU seeking resources pointed out that “I used to learn these things when I was in the MBA, the courses were very theoretical and not practical.” This view is also supported by S2, an undergraduate student in finance preparing business in music”: The financial courses I studied didn't help much, the courses were too theoretical.” The comments of preceding students highlight that professional entrepreneurship courses are too theoretical to guide their projects from practice. However, it is worth noting that some formal courses will bring unexpected resources useful to the entrepreneurs. S2 continued to point out the importance of entrepreneurial resources provided by the formal courses: “But some of the music courses I attended were helpful. Many famous singers in the music industry gave us lessons, like Rae Sremmurd (American singers), they will come to tell us how to make music.” Similarly, students mentioned that they pay more attention to the resources brought by courses compared with the knowledge imparted in formal courses. S1, an undergraduate student in language and computer interested in Internet sales explained that: “The entrepreneurial course is useless. My boyfriend studied MBA courses at AU, he also holds the same view. The reason he went to MBA was to get to know more people and expanding his circle.” In this study, networks tended to play the key role of entrepreneurship capital as social capital for young entrepreneurs starting their businesses in a manner similar to the way Hayter et al. (2016) described networks as resources of entrepreneurship.

Contrary to formal courses, extracurricular activities play an extremely important role in the entrepreneurial engagement of students. It is safe to say that nearly every student expressed their affirmation of extracurricular activities in helping their participation in entrepreneurship, including offering guidance regarding entrepreneurship issues, stimulating the entrepreneurial interest of students, and bringing entrepreneurial resources. The following students all offered insight into the importance they place on the role of extracurricular activities in supporting their start-ups or entrepreneurial demands:

We got the help of Startup in a Box, which has a lot of AU alumni who told us about the stage of the startup and gave us some guidance. And we started to raise money from venture capital, but it is difficult to raise money. AU Technology Development Group gave us some technical support and told us how to raise money. In 2016, we started focus on our startup, and we also got the workspace from the incubator of California Nano Systems Institute at AU.

S5, a post-doctoral student entrepreneur in *material engineering*

I will participate in many activities, like today's WSGR training bootcamp, as well as workshops of Startup Labs. I also go to some lectures of Startup AU. I am building a team now, and I know neither the whole process of starting a business nor how to write a business plan. So, I am actively participating in various activities.

S8, an undergraduate student preparing for business in *mathematics*

I was in the Anderson departments, I did a lot of activities, I went to a lot of these events, it is really nice to meet all of the entrepreneurs that taught me how to start with the business.

*P1*, AU alumni and entrepreneur

I just founded an animation studio, the problem is that I need a lot of advisers you know, how we goanna build it. Because I have the funding already, the problem is I must keep giving project, giving project for back up those funding.

*S6*, a doctoral student in Economics from another university in Los Angeles who is preparing movie and TV animation project

These students showed that extracurricular activities can provide opportunities and channels for students at all stages of entrepreneurship to obtain inspiration, guidance, or resources. These also echo the point of view that several pedagogical methodologies for entrepreneurship education such as lectures were gradually replaced by active methodologies (Peterman and Kennedy, 2003; Fayolle et al., 2006; Heinonen and Poikkijok, 2006; Carvalho et al., 2010). Students are more inclined to participate in various forms of extracurricular activities. The extracurricular activities that AU students participated on the campus include the following: entrepreneurial conferences and forums, coaching, seminars, project development, entrepreneurship boot camp, incubators and accelerators, pitch competition, and Demo Day. The main providers of these activities are the School of Management, AU Technology Development Group, Startup AU, AU Entrepreneurs, AU Business Science Center, etc., as well as a few off-campus organizations.

### Networks as Bridging Assets

The network emphasizes the interaction in the ecosystem, that is, how the elements in the ecosystem are connected (Alvedalen and Boschma, 2017; Motoyama and Knowlton, 2017; Shwetzer et al., 2019). Through the network, individuals or organizations inside and outside the university can effectively communicate, cooperate, strengthen information exchange, share resources efficiently, and allocate resources. The networks can be divided into four categories based on the geographic coverage of the networks, namely, interpersonal network, campus network, regional network (Wang and Ye, 2018), and national or global network.

#### Interpersonal Network

The EE should focus on bridge assets (Mason and Brown, 2014), mainly individuals, that connect people, ideas, and resources (Hechavarria and Ingram, 2014). When the tutors or guests were asked about the reasons for participating in the entrepreneurial activities of AU, five of them said that they came to AU because they know the host/project leader/speaker of the event. As IP nodes, these individuals consciously or unconsciously participate in an interpersonal business network. No participant or company can manage this network, but everyone can influence the network through their communication, expectation, and contribution (Daniel et al., 2018).

#### Campus Network

The entrepreneurial organizations on the AU campus are not isolated, and they are constantly interacting and cooperating to make effective use of resources. For example, the students of AU entrepreneurs use the workspace of start-up AU. The AU Business Science Center collaborated with the investors of AU, Intellectual Property Office, and Corporate Sponsored Research to create an entrepreneurship program in AU Business Science Center. This program transforms and commercializes laboratory technology by combining students and mentors from the fields of medicine, life sciences, physical sciences, engineering, business, and law. The campus network has spawned many entrepreneurship programs and cooperation so far.

#### Regional Network

Hayter et al. (2016) showed that access to external resources and the connection between graduate students and individuals outside the university are necessary for the success of a new venture. A regional network enables entrepreneurs to obtain resources such as knowledge, finance, and human resources (Alvedalen and Boschma, 2017). In fact, the entrepreneurs or the organizers tend to seek resources through the regional network. P14, a pitch competition organizer revealed that he found guests through the local Internet. It is worth noting that this organizer found the guests randomly, and it seems that we can find clues to the impact of spillover of the regional EE on the UEE. Studies have pointed out that the system is connected with other systems and is part of other systems. These parts directly or indirectly affect and are affected by other elements in the system (Sherwood, 2002; Meadows and Wright, 2008; Daniel et al., 2018). When interviewing entrepreneurs in the Los Angeles area, almost all the entrepreneurs interviewed (E1, E2, E3, E5, E6, E7, and P7) reported that Silicon Valley is moving to Silicon Beach in Los Angeles. When a successful start-up company grows to an extraordinary scale and creates significant wealth, it will create a spillover effect in terms of angel investors, venture capitalists, board members, advisors, and mentors (Isenberg, 2010; Hechavarria and Ingram, 2014). It is certain that large-scale company migration is bound to promote the growth of the local entrepreneurial ecology, including the UEE. And this has been confirmed by P10, event specialist of legal firm invited as a guest lecture:

Yes, I agree (that the Silicon Valley is moving to Silicon Beach), the ecosystem in LA is growing these years, the ventures come here, the investors come here, and a lot of company move to Silicon Beach.

In another case, as a key incubation center of AU that benefits entrepreneurs on the campus, the California NanoSystems Institute at AU is also a subproject of the California Institutes for Science and Innovation, which establishes four forms of project centers in the UC System. These regional networks have brought resources to AU entrepreneurial activities and have increased the diversity and richness of AU campus entrepreneurship activities.

#### National or Global Network

The national or global network that has greater energy can nourish individual entrepreneurs and even entrepreneurial organizations. S5, a post-doctoral student entrepreneur, reported that she got support from Y Combinator, the largest seed start-up accelerator in the US. While P3, one of the incubator CEO of AU, reveals that they received funding from the Blackstone Charity Foundation, a charity organization that promotes entrepreneurship and employment growth in the United States:

We are a Blackstone Launchpad program because they are the one who fund our program, the model we are follow is their model.

Global entrepreneurial organizations tend to provide various entrepreneurial resources for universities through the commitment of their members to organizational missions. For example, as described by P9, a guest lecture:

The reason why I came to this event is that today's meeting focus on women's entrepreneurship. The organizer of the meeting today is my business partner. She is also the CFO of Women in Games International which is non-profit. We are committed to setting up our associations all over the world.

In addition to the organizational mission, mutual benefits are more in line with the principles of resource and material exchange within the ecosystem. And this has been commented by P10, event specialist of legal firm invited as a guest lecture:

We often give such public welfare lectures like today, we also do like two days' intensive incubator, like today. Today's event is around half a day, we can get benefit from it, the clients (the student entrepreneurs) today, they might need legal consulting in the future. We not only do this in AU, we also fly like every 1 or 2 month to a lot university, like University of Chicago, Harvard University, Columbia University. Our headquarter is in San Francisco, so we do a lot of mentor there, and in LA we also went to USC, we can say that we do this for nothing, it's like mutual benefits.

### Entrepreneurial Culture Promotes the Agglomeration of Individuals in the EE

Culture is a process of collective thinking that distinguishes members of one group from another (Hofstede, 2001). The cultural impact on the development of the EE cannot be ignored (Suryanto, 2019). Entrepreneurship culture can cultivate the positive attitudes of people toward entrepreneurship and shape entrepreneurial behavior (Krueger et al., 2013; Liñán et al., 2015). National culture, beliefs, motives, values, and personal traits constitute a wide range of themes of entrepreneurial culture (Hayton and Cacciotti, 2013). We found that entrepreneurship values and spirit, entrepreneurial environment, and networking culture are important factors that lead to individual entrepreneurial behavior and promote their agglomeration in the UEE.

#### Entrepreneurial Values and Spirit

Socially shared entrepreneurial beliefs and values could facilitate entrepreneurial actions (Hofstede, 1980; Herbig, 1994; Hayton et al., 2002; Hayton and Cacciotti, 2013). Independence is the entrepreneurial spirit revealed by S1, an undergraduate student in language and computer: “I want to be my own boss, I don't like to work for others and make money for others.” Similarly, Stewart et al. (2003) proposed that achievement motivation is another important cultural variant in entrepreneurship. S2, an undergraduate in finance, directly pointed out the reason he started his business: “I will make a lot of money, and I will be successful in the future.” While some student entrepreneurs have demonstrated the characteristics of social entrepreneurs, that is, solving a certain social problem that motivated their action in entrepreneurship. As explained by S11, an undergraduate entrepreneur in business:

I do not start business for money, because if you start a business just for money, you usually cannot hold on for too long. The reason why I want to start a business is that I have the passion for starting a business, and I really want to solve the social problems. Just like my mobile phone with a broken screen, I am willing to buy a mobile phone screen from China, take it down piece by piece by myself, and install the phone screen. I enjoy the process of solving the problem.

Unlike students, entrepreneurial mentors show different entrepreneurial spirits. Dedication is an entrepreneurial spirit frequently demonstrated by the interviewed mentors. When asked about the reasons why entrepreneurial mentors came to AU and help students start their own businesses, many of them said that they enjoyed the process and hoped that they could help the students start their businesses. As E3, an entrepreneur and mentor commented:

This is because I want to help them, there is no special reason, I want them to start their own business through my suggestion.

P4, an AU alumni and Campus Project Coordinator, showed different reasons for helping the students in entrepreneurship and made it clear as follows:

I am an entrepreneur myself, when I was a student in AU, there were no entrepreneurship programs, and I always wish that there were, so to me it is a great way to give back and help other students, give them support I did not get when I was here.

In addition to dedication, the faith and enthusiasm for entrepreneurship have prompted some entrepreneurs to join EE. As indicated by P11, a guest lecture:

I like to be with these young people who have ideas and creativity. I want to know more about the projects these young people do and how do they do it. I am a believer in entrepreneurs and entrepreneurs, and I believe that entrepreneurship can promote economic development.

#### Entrepreneurial Environment

The entrepreneurial environment surrounding the students on the campus may come from peers or family, which encourages students to have the willingness to start a business on the campus. Entrepreneurial behavior or suggestions from peers can play an exemplary or driving role in the entrepreneurial behavior of students. S9, an undergraduate in computer, touched on this point:

It was interesting. My friend and I developed a software and uploaded it to the Internet. It turned out that some people downloaded it. Then my friend joked: “Why don't we start a business with this?” As a result, we started a business.

S24, a postdoc in material engineering, starting a company, revealed the support they got from the peer influence during the process of starting a business which helped them go through the hard times:

AU did a lot, gave us the basic campus, but the automatically support is that being surrounded by the other people with similar situation, which is very important, it is psychological challenge, the scale like the, the successful mental failure when start up your company, so just be under the people who are all facing similar states, it can help you normalized it, and then you just focus on that and do what you should do.

In addition, some of the campus entrepreneurs indicated that their family has an impact on their entrepreneurial behavior. Family can bring money, networking, and other resources for their success. S7, an alumnus back to AU seeking resources through participating in the activities, indicated the reason for choosing real estate business:

May be the idea was from my dad, he is interested in real estate and always talks about it and gave me inspiration. Besides, the real estate industry can make money.

Interestingly, P12, a guest lecture and incubator CEO, reinforced this point from the angle of the parent when explaining the reason for participating in the event:

I enjoy this process very much. I want to see the success of more people. At the same time, I can also help with my son. My son is starting a business. I want to help my son expand his social network and get more resources through these events.

#### Networking Culture

Networking culture has also played an important role in the formation of entrepreneurial culture of AU. Different from China, almost all the events on the campus have a superbly designed networking part, such as “network break,” “network lunch,” and “networking.” Students are free to communicate, consult, and establish contacts with angels, venture capitalists, corporate executives, financial advisors, and other experts during networking. In this way, they can improve their entrepreneurship programs, get an answer to their doubts, and establish contacts with investors and experts.

### Leadership Plays a Leading Role in Developing the UEE

The establishment and development of the UEE require long-term commitments from university leaders and institutions. These commitments include stable financial resources, and design and establishment of appropriate organizational structures and infrastructures (Art, 2018). The formation process of the EE of AU can provide evidence for this. In 2010, a new vice-chancellor of AU, who is an advocate for promoting research and student entrepreneurship, was appointed. After taking office, the vice-chancellor committed to promote the development of AU entrepreneurship activities, and he has had an important impact on the formation of the AU entrepreneurship ecosystem. Efforts have been made from the following aspects:

To understand the conditions and achievements of the entrepreneurship of students, the vice-chancellor found a famous scholar in the field of management from the School of Management to conduct research on entrepreneurship. Then, this scholar formed a research team and conducted more than 100 interviews in famous American universities such as Stanford University, Columbia University, University of Wisconsin, California Institute of Technology, and so on. Subsequently, three reports were formed which proposed to build an EE in AU and advocate to carry out entrepreneurship activities on the campus. In addition, these reports specifically address the problems of the EE of AU and propose strategies for the future. Subsequently, the vice-chancellor proposed five strategies for developing the entrepreneurial ecosystem in AU, including: (1) establish a proof-of-concept funding mechanism; (2) promote the technical development of campus incubators; (3) targeted investment in the campus technology platform; (4) conduct resident entrepreneurship programs to provide counseling for teachers and students to start businesses; and (5) establish a business advisory committee.

Under the guidance of these strategies, many entrepreneurial organizations have been built to promote knowledge transfer and entrepreneurship. Based on the longitudinal data, we built a tool timeline that reveals the dynamics of the entrepreneurial organizations and their roles developed in AU, as shown in Table 2.

**Table 2:** Timeline of the establishment of the entrepreneurship organization in AU.

**Year**	**Tools**	**Role**
2012	Appointed a new Associate Vice Chancellor for Research and Executive Director of Entrepreneurship	Adjusting the school structure to ensure the promotion of knowledge and entrepreneurship.
2012	Establishment of AU Entrepreneurs	Catering to students' need in every stage of entrepreneurship
2012	Establishment of bVentures (the later Startup Lab)	Provide incubator, accelerator or early stage investor services for technology startups on the campus.
2012	Establishment of Innovation Lab	Promote the transformation of scientific research and technology through the cooperation of multiple disciplines.
2012	Establishment of Startup AU	Aim to create an entrepreneurship culture on the campus and make a connection of students and alumni in entrepreneurship which can help the students entrepreneurs develop and launch their early stage ideas.
2012	Startup AU Accelerator started	Ten-week program provides the early-stage startups with workspace, guidance, mentors and legal services.
2014	Establish Westwood Technology Transfer	Focusing on optimizing and protecting the research discoveries and inventions.
2017	AU Innovation Fund Launched	A proof-of-concept program designed to facilitate the commercialization of advanced AU-owned technologies.

## Discussion

Central to this study is the conviction that extracurricular activities, networks, entrepreneurial culture, and leadership are important factors in developing the UEE. We make this assumption primarily based on the existing research in the UEE. Our study provides empirical evidence and reinforces this point of view relative to the UEE at an AU.

More specifically, we found that, compared with formal courses, students tend to give more positive comments on extracurricular activities. The inability of entrepreneurship courses to function effectively may be attributed to the insufficiency of the entrepreneurship curriculum. Except for business schools, university liberal education cannot provide students with systematic and effective entrepreneurship education and guidance. Here, we considered the study by Art (2018) on universities and EE that UBEEs can range from less to more developed UBEEs. In the embryonic stage of the EE, the UBEE can only provide limited offerings, such as limited courses or student clubs.

Consistent with Art (2018), the network plays an important role in building the UBEE. In other words, the university serves as a bridge to connect the campus and the social community as well as some entity organizations that range from regional to national or global entrepreneurship groups. As important bridging assets, networks promote the interaction and cooperation among EE elements. In these networks, information is transmitted through formal or informal channels, which enable the entrepreneurs to find entrepreneurial opportunities, cofounders, and mentors, obtain entrepreneurial funds, recruit early team members, and share knowledge and other resources.

Culture is an important factor to promote an individual behavior. It will make it easy to understand why these individuals agglomerate in the UEE from a cultural perspective. In this study, many businessmen come to the UEE due to the culture of helping others and back feeding, while student participants are more influenced by the entrepreneurship and surrounding environment.

EEs are intentional communities of economic actors whose individual business activities can largely determine the fate of the EE (Moore, 2006; Daniel et al., 2018). The EE itself may be seen as an intervention tool to mediate the actions of entrepreneurs to achieve specific goals. Thus, politically motivated actors can take action to change the trajectory of the ecosystem (Daniel et al., 2018). A clear vision and institutional support provided by the senior manager of the university helps to implement the entrepreneurial strategy (Clark, 1998). However, it is worth noting that although leadership can play a positive role in the formation of the UEE, but it does not guarantee its formation. The formation of the UEE needs the support of many factors, such as active entrepreneurial culture, developed regional economy, and supportive entrepreneurship policy. It can be safe to say that it is difficult to form the UEE in a second-class university with poor scientific research ability in an underdeveloped area as the UEEs often occur in knowledge-based institutions or regions that have large numbers of scientists and engineers.

In brief, the UEE is a network system with complex elements, including individuals (e.g., student entrepreneurs, faculty, university leaders, project organizers, entrepreneurs, businessmen, venture capitalists, lawyers, consultants, etc.), organizations, curricular activities, extracurricular activities, and capital. Among them, the organizer of the extracurricular activities is the key to connect campus entrepreneurs with resources, as an intermediary, playing the role of connection and service. Entrepreneurship resource providers bring resources for the entrepreneurial activities of students, including entrepreneurs, sponsors, bankers, economists, legal advisers, corporate executives, investors, and other stakeholders. Diversified extracurricular activities have played an important role in stimulating the interest of students in entrepreneurship, providing students with a large number of entrepreneurial resources. Student entrepreneurs also tend to obtain the guidance and resources they need through these activities. All these elements are connected by networks, and resources are circulated through these networks. In addition, the creation of a UEE requires the strong leadership of university leaders. It can be certain that the UEE cannot be built by one side, it is necessary to form multi-side cooperation from the public and private sectors.

Undoubtedly, the factors that affect the UEE are not only the four factors identified by this study. Entrepreneurship courses, national economic policies, innovation and scientific research, venture capital, technology transformation, regional economic development, and entrepreneurial ecology can also be the factors that affect the UEE. The complexity of the UEE makes it impossible for this study to construct its framework completely and include all its influencing factors. Due to space limitations, this exploratory study presents these four factors as the major findings of this research.

## Conclusion

This study was conducted in an American university. Like other qualitative studies, the generalizability of the research findings is limited. The strength of qualitative research is arguably the ability to more deeply contextualize sociological phenomena (Sun, 2018), and this is different from the quantitative research that conducts through a large amount of statistical data. Employing interactive interviews, this study determined the four factors influencing the formation and operation of the UEE through empirical experience, which provides a research foundation for subsequent quantitative research. In the future, it needs to expand the cases by studying the UEE at other entrepreneurial universities worldwide. It is possible that the findings identified in this study may differ in some significant way if conducted at another university, but this does not decrease the value and quality of this research as more factors and mechanisms that affect the formation and operation of the UEE need to be verified to provide more empirical support for future theoretical construction.

Time is a principle that must be adhered to in the research of the UEE. The static method used in studying the UEE neglected the origin and self-sustainable development process of the EE (Mason and Brown, 2014). As UEE has been in the process of development, the temporal nature of the EE is a topic that cannot be ignored (Chou and Zolkiewski, 2012; Möller, 2013; Medlin and Törnroos, 2015; Andersen and Medlin, 2016; Fonfara et al., 2016; Daniel et al., 2018). This study is carried out in a period, so it can only provide insights on one stage of the development of the UEE. The development of the ecosystem will change with time and experience the process of emergence, development, maturity, and even extinction. Therefore, time should be an important dimension when researching or building a UEE in the future.

It should be noted that technological innovation is a feature of entrepreneurship of university students and an indispensable force in the UEEs. Therefore, the impact of organizations, such as incubators and technology transfer offices, on the formation and development of the UEE is an important direction in the future. In addition, the elements of the ecosystem that are conducive to student entrepreneurship are not necessarily the elements of the EE that are conducive to teacher entrepreneurship (Scgaeffer and Matt, 2016; Matt and Scgaeffer, 2018). In fact, this study explains the elements and the role these elements play in the formation and operation of the UEE from the perspective of student entrepreneurs. The role of teacher entrepreneurs in the formation of the UEE is also a direction that needs to be concerned in the future.

## Data Availability Statement

The original contributions generated for the study are included in the article, further inquiries can be directed to the corresponding author.

## Author Contributions

XW designed the study, collected and analyzed data, and wrote the manuscript. SL provided suggestions for the article and helped revise the article. XS and CM provided idea. All authors contributed to the article and approved the submitted version.

## Funding

This research was supported by Scientific Research Start-up Funding Project of Hangzhou Normal University (RWSK20181012), Educational science planning project of Zhejiang Province (2019SCG017).

## Conflict of Interest

The authors declare that the research was conducted in the absence of any commercial or financial relationships that could be construed as a potential conflict of interest.

## Publisher's Note

All claims expressed in this article are solely those of the authors and do not necessarily represent those of their affiliated organizations, or those of the publisher, the editors and the reviewers. Any product that may be evaluated in this article, or claim that may be made by its manufacturer, is not guaranteed or endorsed by the publisher.
